# Significant differences in rates of aseptic loosening between two variations of a popular total knee arthroplasty design

**DOI:** 10.1007/s00264-021-05151-w

**Published:** 2021-08-15

**Authors:** Michael Brown, Rohan Ramasubbu, Mark Jenkinson, James Doonan, Mark Blyth, Bryn Jones

**Affiliations:** Department of Trauma and Orthopaedic Surgery, Glasgow Royal Infirmary, 82 Castle Street, Gatehouse Building, Glasgow, G4 0RH UK

**Keywords:** Total knee arthroplasty, Aseptic loosening, NexGen, Revision, Knee

## Abstract

**Purpose:**

The NexGen Legacy Posterior Stabilised (LPS) prosthesis (Zimmer Biomet, Warsaw, IN, USA) has augmentable and non-augmentable tibial baseplate options. We have noted an anecdotal increase in the number of cases requiring early revision for aseptic loosening since adopting the non-augmentable option. The purpose of this study was to ascertain our rates of aseptic tibial loosening for the two implant types within five years of implantation and to investigate the causes for any difference observed.

**Methods:**

A database search was performed for all patients who underwent primary total knee arthroplasty (TKA) using the NexGen LPS between 2009 and 2015. Kaplan–Meier curves were plotted to assess for differences in revision rates between cohorts. We collected and compared data on gender, age, body mass index, component alignment and cement mantle quality as these were factors thought to affect the likelihood of aseptic loosening.

**Results:**

Two thousand one hundred seventy-two TKAs were included with five year follow-up. There were 759 augmentable knees of which 14 were revised and 1413 non-augmentable knees of which 48 were revised. The overall revision rate at five years was 1.84% in the augmentable cohort and 3.4% in the non-augmentable cohort. The revision rate for aseptic loosening was 0.26% in the augmentable group and 1.42% in the non-augmentable group (*p* = 0.0241).

**Conclusions:**

We have identified increased rates of aseptic loosening in non-augmentable components. This highlights the effect that minor implant changes can have on outcomes. We recommend that clinicians remain alert to implant changes and publish their own results when important trends are observed.

## Introduction

Total knee arthroplasty (TKA) remains the gold standard treatment for symptomatic arthritis of the knee and can significantly improve quality of life for the majority of patients; however, there are undoubtedly still problems related to this procedure which remain unresolved [1]. Since the advent of TKA in the 1970s, revision rates have declined due to a combination of refinement of surgical technique, implant design, fixation methods and improved antisepsis. Despite the decrease in revision rates, the absolute numbers of revision procedures continue to increase due to the increasing number of primary procedures [2, 3]. The most common reason cited for revision knee arthroplasty is aseptic loosening of the components, particularly on the tibial side [2, 4].

**Fig. 1:**
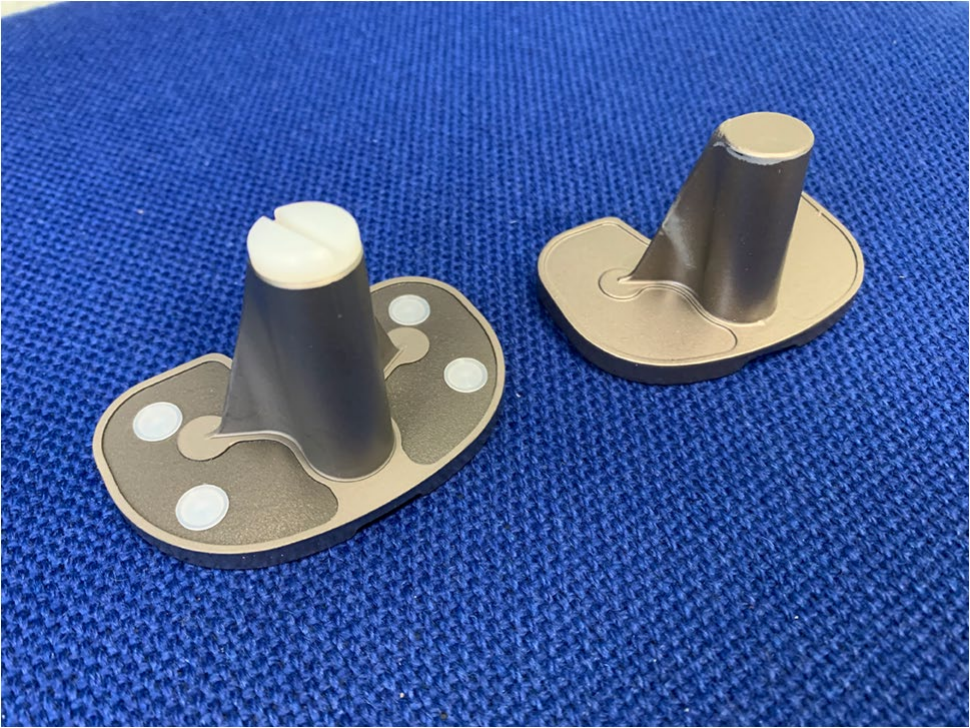
Clinical photograph of an augmentable baseplate (left) and a non-augmentable baseplate (right)

**Fig. 2:**
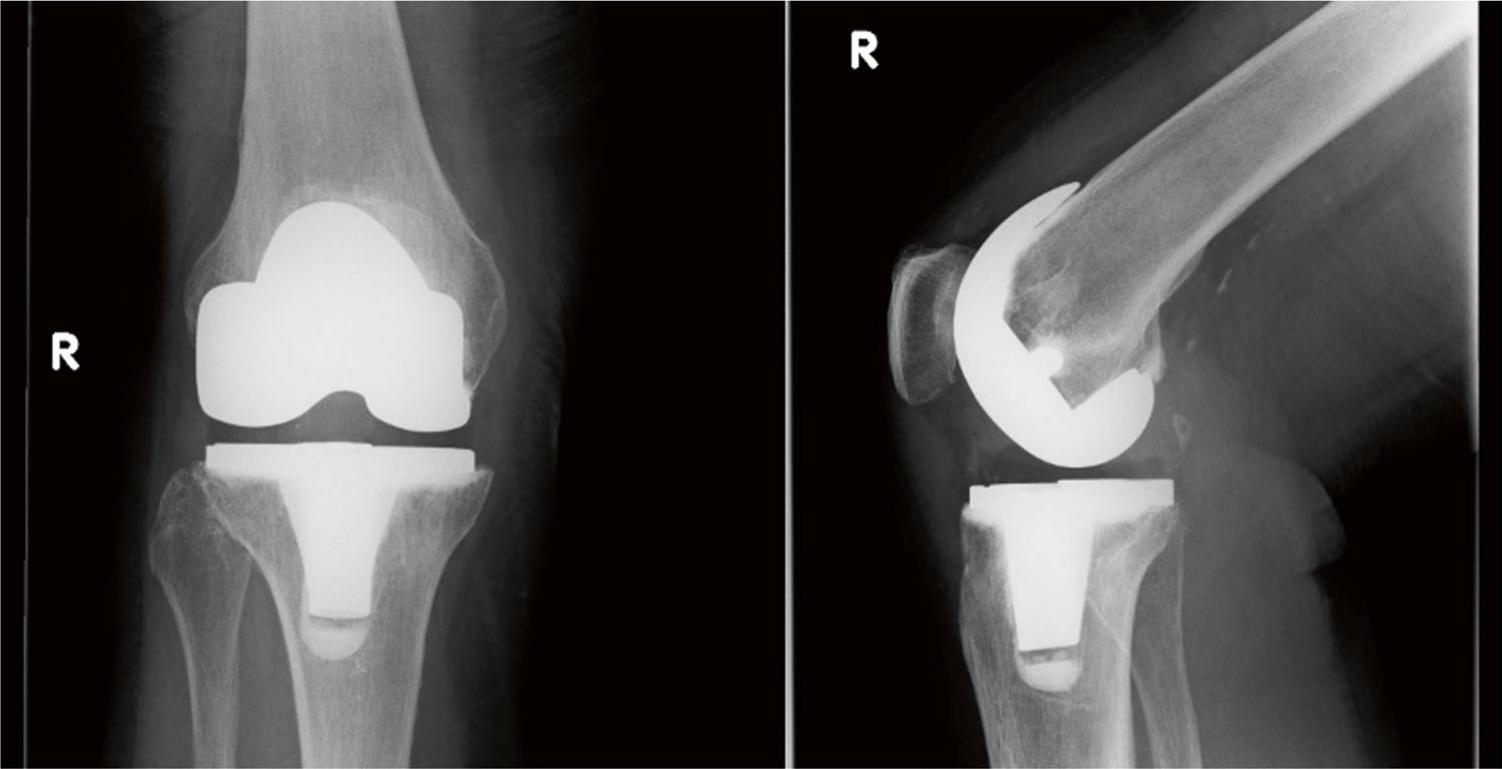
Anteroposterior (AP) and lateral radiographs of an augmentable prosthesis. This component is identifiable by the presence of a radiolucent polyethylene peg at the tip of the keel on the tibial baseplate

**Fig. 3:**
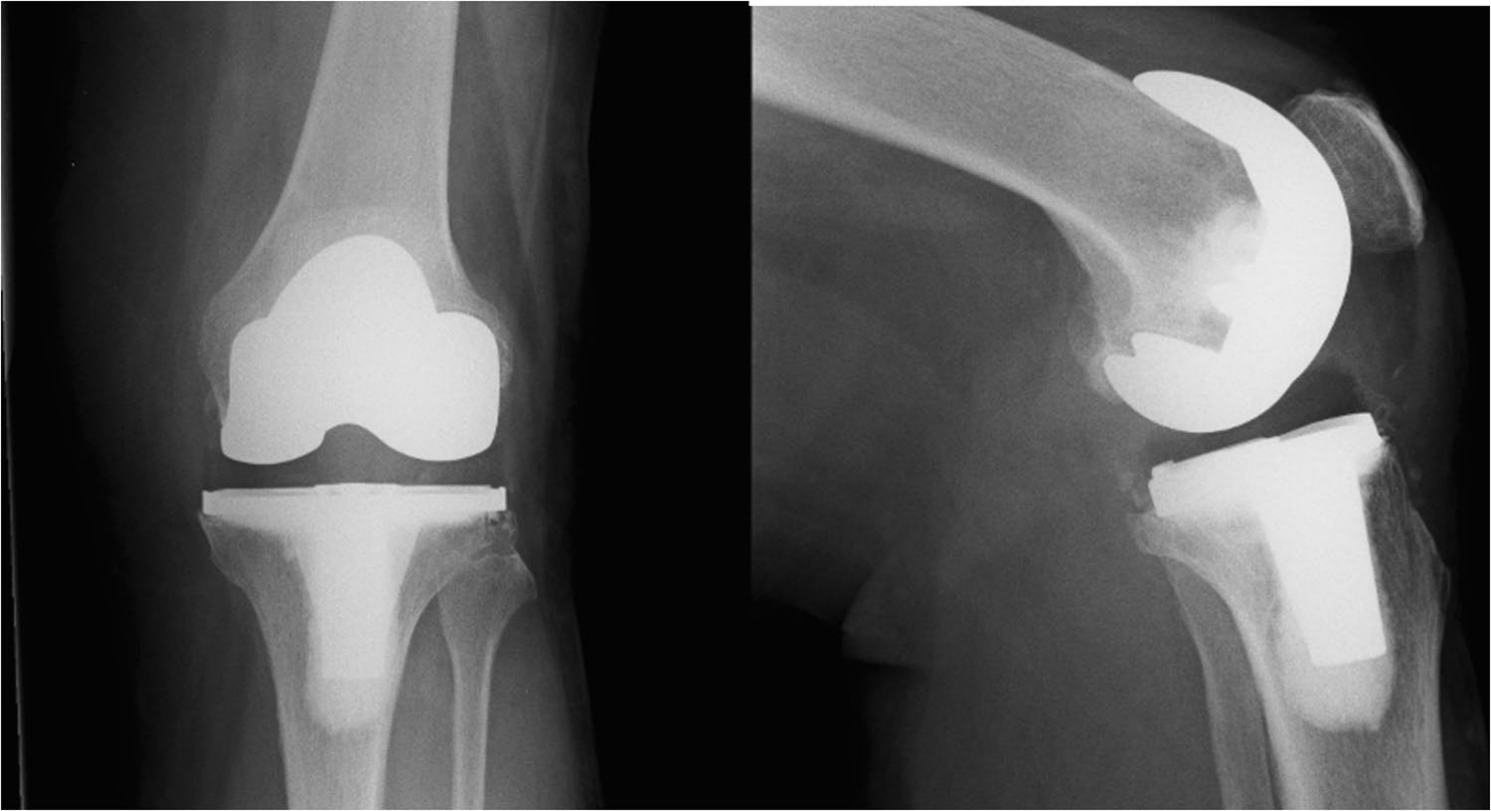
Anteroposterior (AP) and lateral radiographs of a non-augmentable prosthesis. Note the absence of the radiolucent polyethylene plug at the tip of the keel

The NexGen Legacy Posterior Stabilised (LPS) prosthesis (Zimmer Biomet, Warsaw, IN, USA) was introduced to the market in 1997 and has been in use at our unit since 2007 for all primary TKA. This prosthesis has augmentable and non-augmentable options (Fig. 1); the augmentable option features 4 polyethylene lugs on the underside of the baseplate which can be removed to allow for attachment of augments and a polyethylene screw at the tip of the keel which can be replaced by a range of stems should these be required (Fig. 2). The simpler non-augmentable version has neither of these options and is used for the uncomplicated primary TKA (Fig. 3). Baseplates are available with 3° and 7° posterior slope stems. All of the tibial tray options are manufactured using an alloy of Titanium Ti-6-Al-4 V and incorporate a dove-tail locking mechanism for the polyethylene insert [5]. In addition, the NexGen tibial baseplate can be supplied pre-coated with polymethyl methacrylate (PMMA) and a non-pre-coated option is available [6].

Recent joint registry data from the UK reports an all cause revision rate for the NexGen of 2.17% at five years [2]. This includes both cruciate retaining and posterior stabilised designs and also a range of different tibial baseplates. Although registry data continues to become more sophisticated over time, there is insufficient detail within the data to ascertain specific rates of aseptic loosening for different implants within a family of implants at the current time.

Until early 2011, our institution used a 7° augmentable version of the tibial baseplate in all of our primary TKAs in order to keep a consistent inventory and reduce variation. In early 2011, this baseplate was gradually phased out in favour of a lower cost 7° non-augmentable option. At our institution, we have noted an anecdotal increase in the number of cases requiring early revision for aseptic loosening, particularly on the tibial side of the implant. The purpose of this study was to ascertain our observed rates of aseptic tibial loosening for the two implant types within five years of implantation and to investigate the causes for any difference observed. The hypothesis of this study was that there was an increased rate of aseptic tibial loosening in the non-augmentable component compared to the augmentable tibial component as a result of minor differences in the baseplate design.

## Materials and methods

A retrospective search of a prospectively collated Bluespier® (Bluespier International Ltd, Worcestershire, UK) arthroplasty database was performed for all primary total knee arthroplasties (TKAs) performed between 05/01/2009 and 31/03/2015 in our institution with a minimum of five year follow-up. Our regional research and ethics committee were consulted prior to the study but formal ethical approval was not deemed necessary given that it included only a retrospective case note and radiograph review. The number of revision procedures was determined for this cohort during the five year period following implantation and the reason for revision was noted. The case notes for each patient who underwent revision were reviewed to ensure that the coded reason for revision was correct. The Musculoskeletal Infection Society (MSIS) criteria was used to determine whether infection was the cause for revision [7]. Patients were classed as having aseptic loosening if they had symptoms including pain, instability or swelling; had radiographic evidence of loosening; and did not meet the MSIS criteria for infection. Data was collected to indicate the date of revision surgery or date of mortality, age at time of surgery, date of original surgery and body mass index (BMI) for each patient.

We included all patients who underwent primary TKA using the NexGen LPS-fixed bearing prosthesis. All of the implants were posterior stabilised and were cemented without stems or augments. All of the augmentable tibial components had a PMMA pre-coating. The non-augmentable tibial components had no PMMA pre-coating. Palacos® R&G (Heraeus Medical, Hanau, Germany) cement was used until January 2012 until it was phased out over a two month period in order to reduce costs to Refobacin® (Zimmer Biomet, Warsaw, IN, USA). All procedures were performed by, or under the supervision of, one of the nine consultant orthopaedic surgeons within our institution. Over the study period, all surgeons that contributed to this study were high-volume specialists with an interest in knee arthroplasty. Technique for implantation was undertaken as per the operative technique recommended by the manufacturer although there were likely to be subtle differences in techniques utilised by each surgeon.

Post-operative radiographs for the included patients were reviewed to confirm each patient as having an augmentable baseplate (7° fluted stem) or a non-augmentable baseplate (7° fluted stem). An assessment of post-operative alignment and cement mantle quality was performed for all patients who had a non-augmentable baseplate and underwent revision for aseptic loosening. Radiographic views were performed in a standardised fashion by our radiology department but long-leg radiographs were not available. This radiographic assessment was carried out using a standardised technique described by Hampton et al. [8]. Coronal alignment of the femoral and tibial components was measured on the anteroposterior radiograph and sagittal tibial slope was measured on the lateral radiograph. The technique for cement mantle assessment is described in full within the original paper by Hampton et al. and involves counting the number of zones in which there is less than 2 mm of cement-bone penetration, counting radiolucent lines (RLL) and calculating the RLL as a percentage of the surface area available. To facilitate interpretation of these results, we performed the same analysis on a control group of non-augmentable knees which did not go on to require revision and a control group of augmentable knees which did not go on to require revision. The three control groups were selected from the database by searching for patients who were matched for age, BMI and gender as these factors could have an impact on revision rates. For completeness, we also assessed the cement mantle and alignment of the patients who had augmentable baseplates and underwent revision for aseptic loosening although this was a very small group (*n* = 2).

Statistical analysis was performed by an independent researcher using Graphpad Prism version 6. After sorting the primary TKAs into augmentable and non-augmentable cohorts, Kaplan–Meier curves were plotted to assess for differences in all revision rates and aseptic revision rates in both cohorts. Each cohort was also assessed to compare demographics which could potentially affect rates of aseptic loosening. Shapiro–Wilk test was used to determine normality of data and before parametric and non-parametric data was analysed using unpaired *t* tests and Mann–Whitney U tests, respectively. For comparisons containing more than two groups, parametric and non-parametric data was analysed using Fisher’s LSD, one-way ANOVA or Kruskal–Wallis ANOVA with Dunn’s post-test, respectively. Results were deemed significant with a *p* value of < 0.05.

## Results

Overall, 2172 TKAs were performed in the study period with 759 augmentable tibial components inserted between 05/01/2009 and 01/2011 and 1413 non-augmentable tibial components between 01/2011 and 31/03/2015. During this 5-year follow-up, there were 14 revisions in the augmentable group and 48 revisions within the non-augmentable group (Table 1).

**Table 1:** Frequency of indications and percentage of revision surgery following use of augmentable or non-augmentable implants

Indication	Augmentable implant (*n*/%)	Non-augmentable implant (*n*/%)
Infection	8/1.05%	22/1.55%
Stiffness	2/0.26%	3/0.21%
Instability	0/0%	1/0.07%
Aseptic loosening	2/0.26%	20/1.42%
Malalignment	2/0.26%	1/0.07%

There was no difference in BMI or age between either tibial implant group (Table 2) although some data for BMI was missing (augmentable cohort, 445/759; non-augmentable cohort, 769/1413). BMI and age were both recorded at the time of primary surgery. Due to the low patient numbers, it is difficult to draw conclusive comparisons between augmentable and non-augmentable revisions; however, BMI alone does not appear to account for the differences in aseptic revisions observed. Furthermore, it appears non-augmentable aseptic loosening occurs in older patients compared to augmentable loosening although this does not reach statistical significance (*p* = 0.051).

**Table 2:** Age and body mass index for all augmentable and non-augmentable implant patients and revisions indicated by aseptic loosening

	BMI			Age		
	Mean (range)	*n*	*P* value	Mean (range)	*n*	*P* value
Augmentable group	33.0 (17–56)	445	0.058	67.9 (35–89)	759	0.116
Non-augmentable group	32.3 (18–54)	769		68.1 (21–92)	1413	
Augmentable aseptic loosening revisions	40.5 (36–45)	2	0.538	55.5 (55–56)	2	0.051
Non-augmentable aseptic loosening revisions	32.4 (23–41)	20		66.3 (56–79)	20	

**Fig. 4:**
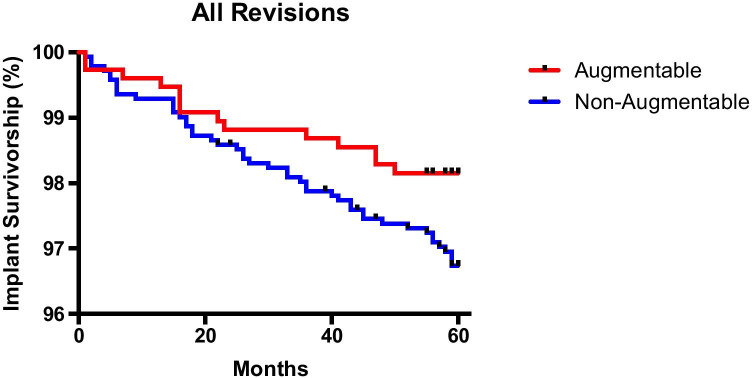
Kaplan–Meier analysis of implant survivorship for 5 years for all cause revisions in the augmentable and non-augmentable implant cohorts (*p* = 0.0562)

**Fig. 5:**
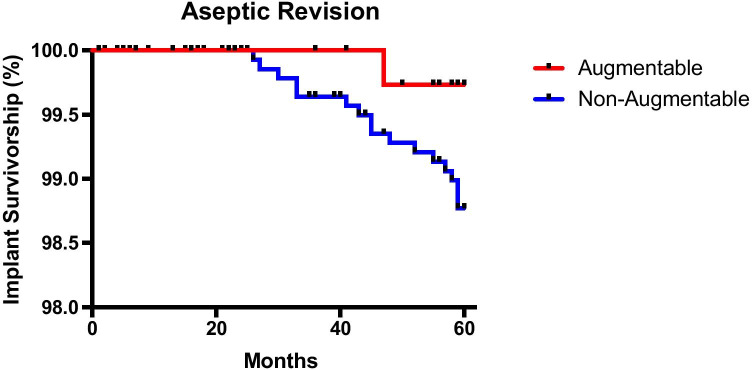
Kaplan–Meier analysis of implant survivorship for aseptic loosening for augmentable and non-augmentable implant cohorts (*p* = 0.024). All other revisions were censored at their revision dates and included in this analysis

The overall all cause revision rate at five years was 1.84% in the augmentable cohort and 3.4% in the non-augmentable cohort. At this same time point, the revision rate for aseptic loosening was 0.26% in the augmentable group and 1.42% in the non-augmentable group. The difference in overall revision rate for the two components was not statistically significant when analysed using the Kaplan–Meier analysis (Fig. 4). However, there was a significant difference (*p* = 0.0241) in implant failure due to aseptic tibial loosening requiring revision between the two cohorts (Fig. 5). The hazard ratio (and confidence intervals) between non-augmentable and augmentable implant is 2.9 (1.1 to 7.5) suggesting that aseptic loosening requiring revision is statistically more likely in the non-augmentable implant cohort than the augmentable cohort.

When assessing post-operative alignment of the non-augmentable group which went on to require revision, there was no significant difference in coronal alignment of the femoral or tibial components when compared to the two control groups. There was however a significant difference in the posterior slope between the aseptic loosening group (5.6°) and the non-augmentable control group (3.2°) (*p* = 0.02) and the augmentable control group (3.4°) (*p* = 0.03) (Table 3).

**Table 3:** Assessment of cementation and alignment for a control group of augmentable knees which did not loosen (*n* = 20), a control group of non-augmentable knees which did not loosen (*n* = 20), non-augmentable knees which required revision for aseptic loosening (*n* = 20) and augmentable knees which required revision for aseptic loosening (*n* = 2)

Variable	Augmentable control, mean (SD)	Non-augmentable control, mean (SD)	Non-augmentable aseptic loosening, mean (SD)	Augmentable aseptic loosening, mean (SD)
Demographic data				
Age	66.5 (7.2)	66.5 (7.5)	66.3 (7.1)	55.5 (0.5)
Female	9	10	10	1
BMI	32.2 (4.6)	32.4 (5)	32.4 (5)	40.5 (6.4)
Component alignment ($^\circ$)				
Femoral component valgus	6.1 (1.8)	5.4 (2.1)	6.2 (2.4)	1.5 (0.7)
Tibial component varus	2.1 (1.8)	1.5 (1.5)	2.6 (1.6)	4 (0)
Tibial component sagittal slope	3.4 (3.2)*	3.2 (3.4)*	5.6 (2.6)	6.5 (3.5)
Measures of cement mantle				
No. of zones cement penetration < 2 mm	1.1 (1.1)	1.7 (1)	2 (1.4)	3.5 (3.5)
No. of RLL at implant cement interface	0.3 (0.8)***	0.8 (0.9)*	1.9 (1.2)	4 (4.2)
No. of RLL at cement–bone interface	0.6 (0.6)	0.7 (0.8)	1.2 (1)	2.5 (0.7)
% surface area RLL compared to surface area available	6.6 (6.6)**	9.9 (9.1)*	19.2 (8.9)	31 (38)

Statistical analysis presented compares control groups to non-augmentable aseptic loosening group where *p < 0.05, **p < 0.01 and ***p < 0.001

Statistical analysis was not performed on the augmentable aseptic loosening group due to the low number of patients

*AP* anteroposterior, *BMI* body mass index, *IC* implant cement, *RLL* radiolucent line, *SD* standard deviation

The cement mantle was assessed (Table 3) and an increased number of RLL were found in the non-augmentable aseptic loosening group (1.9) compared to the non-augmentable control group (0.8) (*p* =  < 0.05) and the augmentable control group (0.3) (*p* =  < 0.001). We also found a higher proportion of RLL as a percentage of the total surface area available in the non-augmentable aseptic loosening group (19.2%) compared to the non-augmentable control group (9.9%) (*p* =  < 0.05) and the augmentable control group (6.6%) (*p* =  < 0.01). There was no significant difference between any of the groups in terms of cement penetration and there was no significant difference between any of the measurements of alignment or cementation when comparing the two control groups. We have not performed statistical analysis on the alignment or cement mantle in the augmentable group aseptic loosening group because the patient numbers were so small (*n* = 2), anecdotally, though one of these knees showed an excellent cement mantle and one had several radiolucent lines.

## Discussion

**Fig. 6:**
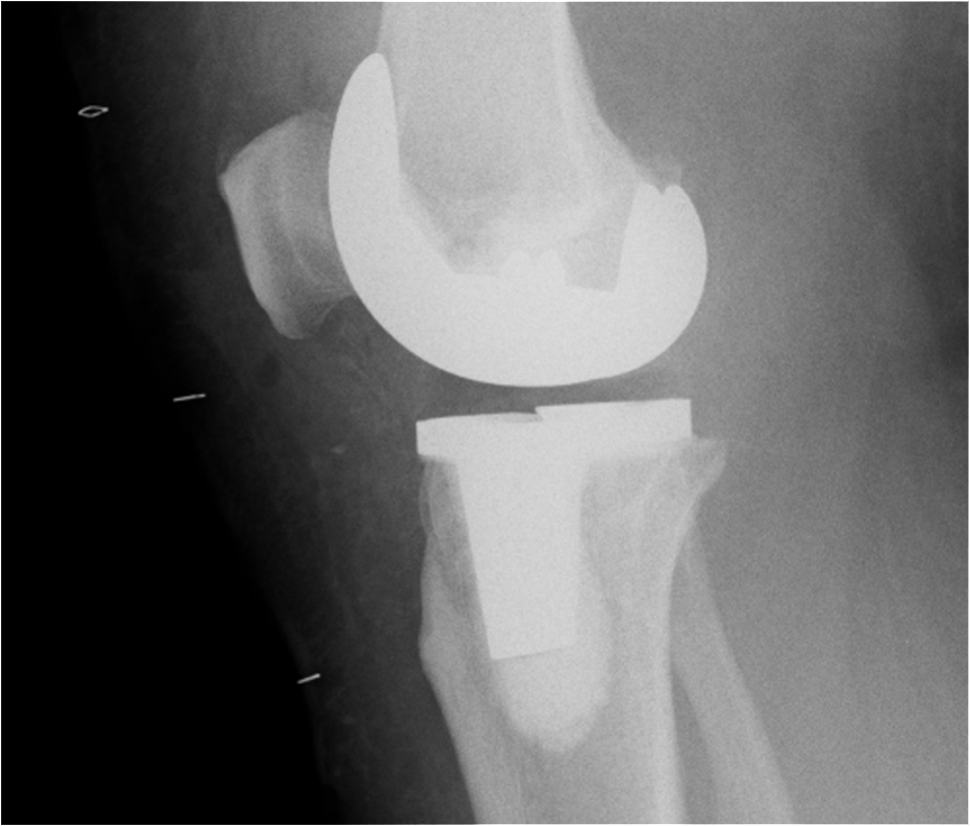
An immediate post-operative lateral radiograph of a non-augmentable TKA. As visible here, we noted the consistent presence of a radiolucent line at the implant/cement interface at the tip of the keel

The purpose of this study was to ascertain differences in rates of aseptic loosening between augmentable and non-augmentable NexGen TKA tibial baseplates used in our unit. We confirmed our hypothesis of increased rates of aseptic loosening in non-augmentable components at five years (*p* = 0.0241) although it may not be possible to confirm that this was solely as a consequence of changes to implant design. There was no significant difference between the cohorts in terms of age or BMI which could account for this difference. We did observe some subtle differences in relation to the quality of the cement mantle in the non-augmentable knees which went on to aseptic loosening. The link between cement mantle quality and risk of aseptic loosening has already been shown [8]. In particular, we noted the consistent appearance of a RLL at the tip of the stem, at the implant cement interface of the non-augmentable components (Fig. 6). In the cases which went on to aseptic loosening, this line seems to progress and failure occurs at the implant cement interface. We observed less posterior slope in the knees which did not require revision comparing to those that went on to loosen aseptically. This was an unexpected finding. We exclusively use the 7° baseplates within our unit because increased posterior slope has been shown to increase post-operative range of flexion [9]. Our intra-operative cutting procedure is in keeping with the operative technique guidance from Zimmer Biomet using an extramedullary jig to match the native tibial alignment and a cutting block to build in the required 7° of posterior slope. It has previously been established that excessive slope leads to flexion instability and even early failure through detensioning of the collateral ligaments [10, 11]. This may offer some explanation for our findings but the overall numbers in the alignment comparison are small so we are reluctant to make any unjustified conclusions in this regard. The increased rates of RLL in the knees which went on to fail suggest that cementation technique may have played a role in the differences observed between our two groups. The effect of the PMMA pre-coating is also difficult to establish definitively. A previous study demonstrated lower rates of aseptic loosening in NexGen tibial baseplates which were non-pre-coated [12]. Conversely, a recent study raised concerns regarding rates of aseptic loosening in non-pre-coated baseplates although there was no direct comparison drawn with the pre-coated version [6]. In that context, it is difficult for us to ascertain the exact effect that the PMMA pre-coating had in our study but we can conclude that it may have contributed to the differences observed between our two groups.

There are several theories on the causes of aseptic loosening. As early as 1976, Harris noted loosening without infection in 4 hip arthroplasties [13]. His initial particle disease theory, which resulted in the development of uncemented implants [14, 15], did not have a positive effect on rates of aseptic loosening, which suggested that the mechanism is likely to be multifactorial. The effective joint space theory was first described in 1992 when it was hypothesised that polyethylene wear particles are dispersed into the effective joint space or that cement particles are generated by debonding or cement fracture [16]. According to this theory, access to the effective joint space is influenced by the cement/implant and cement/bone interfaces. Wear particles activate macrophages which release cytokines involved in bone remodelling such as prostaglandin E2, interleukin 1α, interleukin 6 and tumour necrosis factor α. These cytokines modulate osteoclast and osteoblast activity which results in osteolysis and an increase in the effective joint space [14, 17]. Macrophages may also differentiate into osteoclasts directly to further resorb bone tissue [18]. Stress shielding may also occur in the proximal tibia following total knee arthroplasty. The resultant osteopenia in the bone of the proximal tibia can allow micromotion of the implant which has been shown to result in failure [17]. There is also variation between individuals in the observed rates of osteolysis and it has been proposed that this may be secondary to an adverse cellular response [19]. Exactly, which factors predispose to aseptic loosening is still a matter of debate. Some authors have found an association with post-operative varus malalignment of the tibial component and increased BMI [20, 21]. Others have suggested that aseptic loosening occurs as a direct result of poor cement mantle quality [8]. With cementation being a key factor, some authors suggest that technical factors have the most influence over aseptic loosening rates [4]. But a systematic review was only able to relate an increased risk of aseptic loosening to male gender and high activity levels [22]. There have certainly been high rates of aseptic loosening observed with specific components which has led to a decrease in their usage [23].

Interpretation of our results in comparison to the literature is challenging. As previously noted, a number of different versions of the NexGen tibial baseplate are available, and in some studies, it can be difficult to determine exactly which version was being used [24, 25]. Similar problems are encountered when assessing arthroplasty registry data, and the cause of revision is often not clear [2]. Joint registry data for England and Wales has shown an all cause revision rate for the NexGen implant of 2.17% at five years [2]. This compares to our all cause revision rate of 2.85% at the same time point. The all cause revision rate for the non-augmentable tibial baseplate in our study however was 3.4% compared to a 1.84% rate in the augmentable group. This difference was not statistically significant (*p* = 0.0562). When we consider the rates of revision for aseptic loosening, it is useful to draw comparison to the results obtained by Arsoy et al. in 2013 [5]. In this study with similar methodology to our study, they observed a 2.2% revision rate for aseptic loosening at five years when assessing the 3° augmentable NexGen tibial baseplate. Our results compare favourably with these findings given that we found a revision rate for aseptic loosening of 0.26% at five years for the 7° augmentable baseplate and 1.42% for the non-augmentable 7° baseplate. Whilst our rates of revision for aseptic loosening in both groups compare favourably with previously reported rates, we have demonstrated an increased rate of revision for aseptic loosening at five years in the non-augmentable baseplate compared to the augmentable alternative. We have been unable to explain the difference observed when considering BMI or age but we accept that cementation technique may have played a role.

The strengths of this study include the overall patient numbers and the methodology. We also use the NexGen exclusively within our unit which ensures that all surgeons are familiar with it and there are no low-volume surgeons involved in this study in either time period.

There are a number of weaknesses in the study. When assessing alignment of the non-augmentable knees which failed due to aseptic loosening, we used short knee radiographs. Clearly, the use of long-leg alignment films would have been preferable but these were rarely available and the use of the short knee radiograph has been validated in previous studies [8]. BMI data was incomplete in our database but was present in similar proportions for both cohorts. We recognise that using revision as an endpoint for failure has its shortcomings and is likely to overestimate implant success. Furthermore, there is a small chance that there were some failures who underwent revision out with our local area or in the private sector. Evidence from the Scottish Arthroplasty Project in 2009 showed that the overall numbers of patients moving between health boards were small around the time of our study [26]. Furthermore, patients who undergo revision within Scotland are traced back to the hospital in which they underwent primary surgery when their data is collected for the Scottish Arthroplasty Project. Only patients who underwent revision surgery out with Scotland or within the private sector would have been missed in a national Picture Archiving and Communication System (PACS) search. Refobacin® cement (Zimmer Biomet, Warsaw, IN, USA) was only used in the non-augmentable cohort given the timing of the introduction of this product in our institution. It is possible therefore that this could have contributed to the aseptic loosening rate differences or indeed the radiographic differences within the cement mantle. In 2011 though, we were using non-augmentable baseplates with the original Palacos® R&G (Heraeus Medical, Hanau, Germany) cement. In that year, we found that there were 5 knees which went onto aseptic loosening. The rate of failure of the non-augmentable baseplate did not increase further after the cement was changed although because the overall numbers per year were very small, statistical analysis has not been performed. Previous evidence from the Norwegian arthroplasty register has shown similar survival of TKAs cemented with Palacos versus those cemented with Refobacin [27].

To our knowledge, ours is the first study to compare aseptic loosening rates between these two baseplate types although a recent paper compared rates of aseptic loosening between the NexGen non-modular baseplate and Sigma® P.F.C® TKA (DePuy Synthes, Warsaw, Indiana) and found much higher rates of aseptic loosening of the NexGen tibial baseplate [24]. Whilst our rates of aseptic loosening from both implants are likely to be within what might be considered acceptable limits, the difference observed highlights the effect that minor changes to an implant can have on outcomes in the medium term. With a great deal of heterogeneity within arthroplasty registry data, it is important that clinicians remain alert to seemingly minor implant changes within their own units and publish their own results when important trends are observed. Our regional Zimmer Biomet representative has confirmed that in the last ten years, 78.84% of all primary NexGen knees implanted in the UK were non-augmentable.

In conclusion, we have demonstrated a significantly higher rate of aseptic loosening in the Zimmer NexGen TKA when using a non-augmentable 7° baseplate compared to an augmentable 7° baseplate. Further investigation of the rates of aseptic loosening in the non-augmentable baseplate at other units may be warranted to determine if these findings are replicable or if they may in part be due to the introduction of Refobacin® cement. Given the lower cost of the non-augmentable baseplate, a cost analysis based on the increased rate of revision observed may also be useful in order to consider whether there is any financial benefit in using this implant.

## Data Availability

Not applicable.
